# Variation in Male Mate Choice in *Drosophila melanogaster*

**DOI:** 10.1371/journal.pone.0056299

**Published:** 2013-02-06

**Authors:** Dominic A. Edward, Tracey Chapman

**Affiliations:** School of Biological Sciences, University of East Anglia, Norwich, Norfolk, United Kingdom; North Carolina State University, United States of America

## Abstract

Male mate choice has been reported in the fruit fly, *Drosophila melanogaster*, even though males of this species were previously thought to maximise their fitness by mating with all available females. To understand the evolution of male mate choice it is important to understand variation in male mating preferences. Two studies, using different stock populations and different methods, have reported contrasting patterns of variation in male mate choice in *D. melanogaster*. Two possible explanations are that there are evolved differences in each stock population or that the methods used to measure choice could have biased the results. We investigated these hypotheses here by repeating the methods used in one study in which variable male mate choice was found, using the stock population from the other study in which choice was not variable. The results showed a significant resource-independent male preference for less fecund, smaller females, which contrasts with previous observations of male mate choice. This indicates that different selection pressures between populations have resulted in evolved differences in the expression of male mate choice. It also reveals phenotypic plasticity in male mate choice in response to cues encountered in each choice environment. The results highlight the importance of variation in male mate choice, and of identifying mechanisms in order to understand the evolution of mate choice under varying ecological conditions.

## Introduction

Mate choice occurs whenever traits in one sex increase the probability of mating or reproductive investment with specific individuals of the opposite sex [1], [2]. Though mate choice is most often identified in females, the significance of mate choice in males is increasingly recognised [3]. Male mate choice has been reported in the fruit fly *Drosophila melanogaster* [4], [5], [6] as well as other species of *Drosophila* [7], [8]. These reports are particularly interesting because male mate choice was not originally expected in species where males are anticipated to maximise fitness by mating with all available mates and where males provide no parental care [9], [10]. Nevertheless, male mate choice is potentially favoured in *D. melanogaster* because of constraints on male mating rate arising from ejaculate exhaustion [11], [12].

To understand how male mate choice can evolve in *D. melanogaster*, it is essential that we understand the benefits to a male of choosing between females. Furthermore, insight into the factors associated with variation in male mate choice is key, as this is the variation upon which selection will ultimately act. This is the focus of the investigation of this study.

It has been demonstrated, in two different stock populations, that male *D. melanogaster* preferentially mate with larger, more fecund females [4,5, also see 6]. These studies demonstrate that males can benefit from exercising mate choice and that this benefit can help to maintain the expression of male mate choice. However, these studies also differed in the methods used to measure choice and the amount of variation in male mate choice that was recorded. Male mate choice was first identified by Byrne & Rice in tests using groups of 10 males choosing between 10 large and 10 small females [5]. In contrast, Edward & Chapman measured the mate choice of single males when given a choice between two randomly selected females [4]. Byrne & Rice varied the amount of mating resources available to males by comparing virgin males to males that had recently mated multiple times [5]. Edward & Chapman also tested for plasticity in male mate choice by varying whether males had previously mated, by exposing males to rivals and by varying the larval density at which males were reared. Both studies found that larger, more fecund females were significantly more likely to be mated than smaller, less fecund females. Byrne & Rice found a difference in proportion of large and small females that were mated of between 8% and 15% according to the availability of resources, with resource depleted males being choosier [5]. In a subsequent study, Edward & Chapman found that males would mate with the more fecund female on 54% of occasions and the less fecund female on the remaining 46% of occasions, a difference of 8% [4]. However, Edward & Chapman found no variation in male mate choice under the different conditions. This suggests that male mate choice was more variable in the study conducted by Byrne & Rice [5] than in Edward & Chapman [4].

Variation in the strength and plasticity of mate preferences is potentially very interesting because it could help to reveal different forces of selection maintaining the expression of male mate choice [13]. One explanation for differences in variation in the strength of male mate choice is that Byrne & Rice manipulated variation in female fecundity, whereas Edward & Chapman did not [4], [5]. Byrne & Rice achieved this by rearing flies at high and low larval density. This manipulation of female fecundity is expected to enhance the benefits of being choosy and could facilitate male choice by enhancing phenotypic differences between females [4]. Variation in female fecundity was correlated with female body size, though there may be a host of other, less obvious, phenotypic characters that could also act as cues of female fitness to males. However, there are two further explanations for the differences between these two studies. First, the different methods used in each study to measure choice could have been biased in their ability to detect choice, hence one method may be better at detecting variation in choice. Second, the stock cultures used in these studies may have evolved to express choice in distinct ways because of divergent selection pressures acting in each stock population. Byrne & Rice measured male mate choice in the LH_M_ stock population which is maintained with discrete generations, at controlled population densities and interactions between adults are restricted to a 48 hour period each generation [for details see 14]. In contrast, Edward & Chapman measured male mate choice in a Dahomey stock population that is maintained with overlapping generations and no control of population density or time limits to adult behavioural interactions [4].

The objective of this work was to determine whether the differences found between these two studies is due to differences in the expression of mate choice in each stock population or differences in the protocols used to measure choice. To achieve this objective we replicated the experiment performed by Byrne & Rice [5] using the same stock population as used in Edward & Chapman [4]. As in Byrne & Rice [5], we used males that were resource depleted (mated multiply just prior to the mate choice tests) and non-resource depleted. This was to test for the influence of a male's immediate sexual history upon his propensity for choosiness.

This work is important to help understand how variation in male mate choice occurs and hence how important a contributor it is likely to be overall to sexual selection. In addition, this work demonstrates not only the importance of replicating experiments, but the benefit of replication using different study populations and variation that can arise when different protocols are used to investigate similar hypotheses.

## Materials and Methods

### Experimental Procedure

Flies were obtained from the Dahomey stock, which is a large outbred laboratory population with overlapping generations. Culture and experimental conditions were 25°C and a 12 h:12 h light:dark cycle. Standard culture vials (75 mm height×25 mm diameter) contained 8 ml of standard sugar-yeast food (100 g brewer's yeast, 100 g sucrose, 20 g agar, 30 ml Nipagin (10% w/v solution) and 3 ml propionic acid per 1 L medium). Adult flies were collected as virgins and stored in same sex groups of 10 in standard culture vials that were seeded *ad libitum* with live yeast granules. Flies were aged for at least 48 hours before use in experiments to ensure sexual maturity. The virginity of all females was checked before use in the experiment by examining vials for the presence of larvae.

All males were reared at a density of 150 larvae per standard culture vial. To generate females of large and small body size larval density was manipulated. Females of small body size were reared at 1000 larvae per vial (125 larvae per ml) and of large body size at 50 larvae per vial (6.25 larvae per ml). These larval densities were used as they are similar in magnitude and replicate the relative difference in larval densities used by Byrne & Rice. To establish whether there were the expected fecundity differences between small and large females, we counted the number of eggs and offspring from a sub-sample of mated females allowed to oviposit for 24 hours. Vials were then incubated for 12 days to allow offspring to develop.

Males used in experimental treatments were either ‘non-resource depleted’ males or ‘resource depleted’ males. Resource depleted males were generated by placing 10 males with 40 virgin females into a vial for 5 hours. After this time males were anaesthetised using CO_2_, transferred to a new culture vial and allowed to recover for 1 hour before the experiment. By exposure to an excess of virgin females, ‘resource depleted’ males each mated multiple times before the choice tests. These males therefore had reduced resources available for mating in comparison to non-resource depleted males.

### Mating Controls – Large and Small Female Receptivity

The measurement of male mate choice for large and small females could be biased if females of different sizes exhibited differences in their receptivity. To test for the possibility that small and large females differed in their receptivity to males we ran trials where 10 males were introduced into a vial containing either 20 small females or 20 large females. The procedure used to mate flies and score fertilizations were identical to those used in experimental treatments (below). Mating controls were repeated on two different days. 12 replicates of each mating control were conducted on the first day and 11 replicates of each mating control on the second day.

### Experimental Treatments–Male Mate Choice for Large or Small Females under Resource Depleted and Non-Depleted Conditions

In the mate choice tests, we examined whether males showed a preference for large females. We ran trials where 10 males were introduced into a vial containing 10 small and 10 large females. After 30 minutes flies were anaesthetized using CO_2_ and females were housed individually in standard culture vials. Vials were incubated for 4 days and then examined for the presence of larvae, in order to score whether the female had mated. Experimental treatments were repeated on two different days. 12 replicates were conducted on the first day and 11 replicates on the second day.

### Statistical Analysis

The fecundity of large and small females was compared using Student's t-tests. The proportions of small and large females that had mated in the mating control tests were compared in a General Linear Model with a binomial error distribution. Female size, replicate day and their interaction were included as fixed effects. Data were not found to be overdispersed. Two methods of analysis were performed to assess male preferences for female size under normal and resource depleted conditions. First, we counted the number of vials in which males mated more frequently with either larger or smaller females. These frequencies were compared using a binomial test to examine whether large or small females were mated more frequently. A χ^2^ test was also used to investigate whether resource depleted or non-resource depleted males were more likely to mate with either large or small females. This analysis mirrors that performed by Byrne & Rice, but with the addition of the χ^2^ test to compare different male treatments. To explore in more depth the propensity of males to mate with large versus small females under normal and resource depleted conditions, the proportion of mated females was compared in a Generalised Linear Mixed Model with a binomial error distribution. Female size, male treatment, replicate day and all possible interactions were included as fixed effects and testing vial was included as a random effect. Data were again not found to be overdispersed. The significance of each fixed effect was determined by a likelihood ratio test that compared reduced and full models. All analyses were conducted in R v2.11.1 (R Foundation for Statistical Computing, Vienna, Austria).

## Results

### Fecundity of Small and Large Females

As expected, large females that were reared at low larval density (N = 39) produced significantly more eggs and more offspring than small females reared at high density (N = 36; mean number of eggs±95% confidence interval: large females = 73.38±8.53, small females = 59.25±6.32; t_73_ = 3.649, p<0.001; mean number of offspring±95% confidence interval: large females = 66.51±9.21, small females = 54.39±6.13; t_73_ = 2.112, p = 0.038).

### Mating Controls–Large and Small Female Receptivity

Males inseminated as many large females as small females in the ‘no choice’ mating controls (mean % females inseminated±95% confidence interval; large females = 55.81%±20.30%, small females = 54.29%±20.36%; χ^2^_1_ = 0.202, p = 0.653). The lack of effect of female size on the proportion of females that mated was consistent across replicate days (χ^2^_1_ = 0.609, p = 0.435). Consequently, there was no evidence that receptivity differences between large and small females could have biased the mate choice results reported below. A recent study also showed that the same range of variation in female larval density had not effect on female latency to mate [15].

### Experimental Treatments-Male Mate Choice for Large or Small Females under Resource Depleted and Non-Depleted Conditions

**Figure 1:**
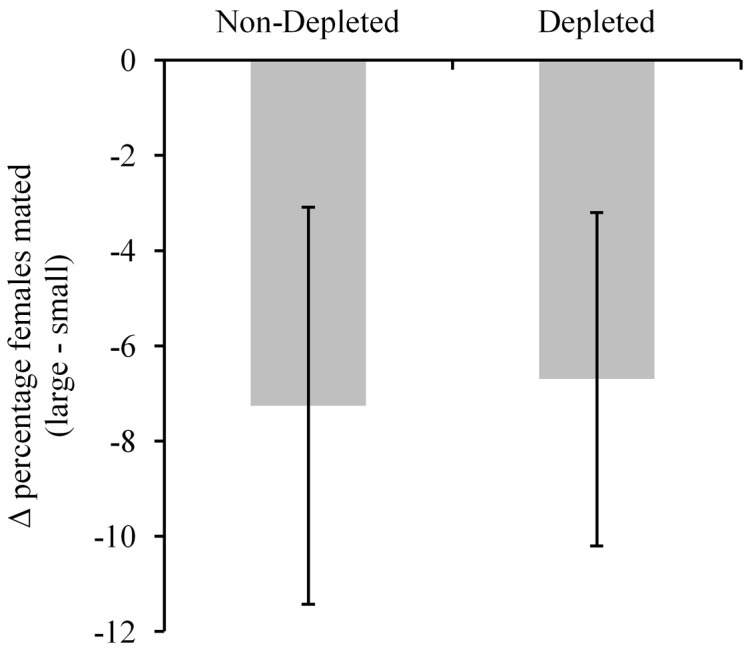
Differences in the percentage of large and small females that were mated. The difference in the percentage of large and small females chosen as mates when males were either resource depleted or not depleted. Resource depleted males had mated multiple before being given a choice of female whilst non-resource depleted males were virgin. (Mean±S.E. of the percentage of large females mated–percentage of small females mated).

In choice tests males preferred to mate with small females. On 33 occasions males mated more frequently with smaller females and on 13 occasions males mated more frequently with larger females (binomial test, p = 0.005). Males in the resource depleted and non-resource depleted groups were both more likely to mate with smaller females than larger females (7 out of 23 vials and 6 out of 23 vials respectively, χ^2^_1_ = 0.107, p = 0.743). Results of the GLMM confirmed that males were significantly more likely to mate small rather than large females (χ^2^_1_ = 4.570, p = 0.033) and this was consistently observed across replicate days (χ^2^_1_ = 0.782, p = 0.377). The bias to mate with small females was not altered by the depletion of male resources (χ^2^_1_ = 0.013, p = 0.909). The propensity of males to mate large versus small females is illustrated by calculating Δ percentage values (% large females mated-% small females mated; Fig. 1).

## Discussion

In this study we measured male mate choice using a novel combination of methods and stocks previously employed in order to gain insight into variation in the expression and potential benefits of male mate choice. We tested choice in the Dahomey stock population of *D. melanogaster* by replicating the protocol used by Byrne & Rice that was previously used to measure male mate choice in another wild type population (the LH_M_ stock) [5]. The objective of this replication was to determine if differences in male mate choice reported by Byrne & Rice and Edward & Chapman could be due to evolved differences in the stock populations used or differences in the protocols used to measure mate choice.

Byrne & Rice observed significant male preference for larger, more fecund females (LH_M_ population), with the magnitude of choice depending on the mating resources/sexual experience of males [5]. In contrast, in our current study we found significant resource-independent male preference for smaller, less fecund females (Dahomey population). Furthermore, the preference of Dahomey males to mate with less fecund females found here is opposite to the preference for more fecund females found previously in the same stock population [4]. From these findings we can infer two conclusions. First, there is a difference in the mate choice expressed by males from the two wild type stock populations. Second, the measurement of mate choice expressed by males from the Dahomey stock population is dependent on the protocol used to measure choice.

It is worth noting that although perhaps the most obvious change in females following development at different larval densities is variation in body size, it is likely that many other phenotypic traits will also vary. One example is that of cuticular hydrocarbon profile [16], which could form the proximate basis for male preferences. Nevertheless, irrespective of the precise trait which males use to discriminate between different females, the ultimate outcome remains unchanged-less fecund females from the high larval density environments were preferred in this study whilst more fecund females from the high larval density environment were preferred in previous work [5].

We first conclude that there is a difference in mate choice expressed by males from the different populations because, when measured using the same experimental procedure, both the direction and resource-dependence of mate choice was different. In addition, the lack of resource dependence of mate choice by Dahomey males is consistent with our previous results [4]. Overall, these findings are consistent with differential selection acting in each of these populations with respect to male mate choice. Differences in selection for male mate choice are likely to have arisen because of the differences in culture conditions. For example, in the LH_M_ population males have a restricted window of opportunity lasting just 48 hours in which they can compete for mates in ‘adult competition vials’ [14]. During this period adult population density is reduced to increase the potential for behavioural interactions to influence individual fitness [14]. In contrast, population density and the period of time in which males have the opportunity to mate are unrestricted in the Dahomey stock. We would expect that these differences will exert different selection pressures for male traits to maximise individual fitness.

However, we also conclude that the measurement of mate choice expressed by males from the Dahomey stock population is dependent on the protocol used to measure choice. This is because we find a preference for small females in this study but a preference for large females in previous work [4]. There are two possible explanations. First, the two measurements of mate choice could represent phenotypic plasticity in male mate choice arising due to the different experimental conditions present in each assay protocol. Second, the measurement of male mate choice observed here could be a pleiotropic effect of measuring choice under conditions that are not normally experienced by males from the Dahomey population.

The first explanation-that the different size preference is the result of phenotypic plasticity in response to assay condition-might seem unlikely given that no plasticity in male mate choice was found previously for this population in response to factors including variation in larval density, exposure to rival males or male mated status [4]. However, one important difference between the choice assay environment used here, following the methods of Byrne & Rice [5], is the number of adult flies present at the time of mate choice. In this study, there was a total of 30 adult flies (10 males and 20 females) present in the choice arena. However, in Edward & Chapman there were only 3 adult flies present (1 male and 2 females). This difference in the social environment could have influenced the expression of choice. The presence of other males in the choice arena could indicate different levels of competition whilst the presence of females could influence male perception of the likelihood of future mating as well as presenting wider variation for choice.

Social environment has been found to influence male mate choice in other species [e.g. 17,18] though in *D. melanogaster* it has been found that exposure to rival males prior to mating does not influence male choice [4]. Nevertheless, exposure to rivals at the time mate choice is expressed could have a very different effect from exposure to rivals prior to choice. For example, mating duration increases when males are exposed to rivals prior to mating but decreases when exposed to rivals at the same time as mating [19]. It is therefore plausible that exposure to rival males simultaneously with the expression of choice could influence male mate choice even though there is no effect of prior exposure to rival males [4]. Interestingly, the study by Long et al. [6] that used the same stock population as Byrne & Rice found a male preference for larger females irrespective of the presence of other adult flies, 16 males choosing between 16 females or 1 male choosing between 2 females. This would suggest that variation in male mate choice caused by the social environment is not present in the LH_M_ stock population. Different responses to social environment could reflect different selection pressures operating in the LH_M_ and Dahomey stocks.

If the male preference for smaller females found in this study is the result of phenotypic plasticity in male preferences due to different selection pressures then an adaptive explanation for preferring smaller, less fecund females is required. One possibility is that smaller individuals develop faster, hence generation time might be reduced for males choosing smaller females. This may be more beneficial to male reproductive fitness than producing a larger number of offspring under some conditions. We also note that the proportionate difference in fecundity between large and small females was lower in this study than that of Byrne & Rice. This would make it more difficult for males to discriminate between females in this study, yet this would not explain why males were found to prefer smaller females.

A second possible explanation for the different female size preferences found in Dahomey males is that the preference for small females found here is simply a pleiotropic effect of measuring mate choice under conditions that are not normally experienced by males from this population. Because the husbandry practices of the two stock populations, LH_M_ and Dahomey, are different and because it is important to measure traits under standardised conditions, the use of the same protocol as designed to measure choice in the LH_M_ population could itself have produced contrasting results. Consequently, an important question is exactly how to compare male mate choice between populations. On the face of it unless the same conditions are used to measure choice the results cannot be directly compared. As the two stocks are both laboratory populations of the same species, it would seem sensible to adopt the same protocols. However, given the differences in mate choice that have been identified through this study a reappraisal of exactly how to compare choosiness between different populations may be needed.

In summary, the results of this work show that using a single approach to measure mate choice in a single population of an organism may not be representative because mate choice can vary considerably between populations and under different conditions. Because of their different evolutionary histories, different populations may express different patterns of choice that may vary in different ecological settings. Though this has been highlighted here in a laboratory-adapted organism, this result is highly pertinent to wild derived populations of species where ecological variation is greatly magnified. We show that differences between studies, rather than presenting obstacles to the study of mate choice, instead present valuable opportunities to investigate the underlying causes of variation in mate choice.
